# The anti-neoplastic activity of Vandetanib against high-risk medulloblastoma variants is profoundly enhanced by additional PI3K inhibition

**DOI:** 10.18632/oncotarget.14911

**Published:** 2017-01-31

**Authors:** Rogerio B Craveiro, Michael Ehrhardt, Julia Velz, Martin Olschewski, Barbara Goetz, Torsten Pietsch, Dagmar Dilloo

**Affiliations:** ^1^ Department of Pediatric Hematology and Oncology, Center for Pediatrics, University of Bonn Medical Center, D-53113 Bonn, Germany; ^2^ Department of Neuropathology, University of Bonn, D-53105 Bonn, Germany

**Keywords:** medulloblastoma, Vandetanib, GDC-0941, targeted therapy, multi-kinase inhibitor (MKI)

## Abstract

Medulloblastoma is comprised of at least four molecular subgroups with distinct clinical outcome (WHO classification 2016). SHH-TP53-mutated as well as MYC-amplified Non-WNT/Non-SHH medulloblastoma show the worst prognosis.

Here we present evidence that single application of the multi-kinase inhibitor Vandetanib displays anti-neoplastic efficacy against cell lines derived from high-risk SHH-TP53-mutated and MYC-amplified Non-WNT/Non-SHH medulloblastoma. The narrow target spectrum of Vandetanib along with a favourable toxicity profile renders this drug ideal for multimodal treatment approaches. In this context our investigation documents that Vandetanib in combination with the clinically available PI3K inhibitor GDC-0941 leads to enhanced cytotoxicity against MYC-amplified and SHH-TP53-mutated medulloblastoma. In line with these findings we show for MYC-amplified medulloblastoma a profound reduction in activity of the oncogenes STAT3 and AKT. Furthermore, we document that Vandetanib and the standard chemotherapeutic Etoposide display additive anti-neoplastic efficacy in the investigated medulloblastoma cell lines that could be further enhanced by PI3K inhibition. Of note, the combination of Vandetanib, GDC-0941 and Etoposide results in MYC-amplified and SHH-TP53-mutated cell lines in complete loss of cell viability. Our findings therefore provide a rational to further evaluate Vandetanib in combination with PI3K inhibitors as well as standard chemotherapeutics *in vivo* for the treatment of most aggressive medulloblastoma variants.

## INTRODUCTION

Angiogenesis is critical for tumor growth and metastasis, therefore receptor tyrosine kinase inhibitors targeting tumor blood vessel formation by VEGFR inhibition increasingly gain access to multimodal therapeutic approaches against brain tumors [1, 2]. Recent clinical studies in adult and pediatric patients suffering from glioblastoma multiforme and diffuse pontine glioma document anti-neoplastic activity within the central nervous system and good tolerability of Vandetanib in single use or in combination with radio- and chemotherapy [3–5]. Against this background our study evaluates the potency of Vandetanib for medulloblastoma treatment, the most common malignant brain tumor of childhood. Numerous molecular studies document that medulloblastoma is not one disease but composed of molecular distinct subgroups [6]. Overall survival for patients suffering from SHH-TP53 mutated and MYC-amplified Non-WNT/Non-SHH medulloblastoma subtypes is poor with the highest risk of recurrence and death within this patient cohort [7, 8]

The efficacy of Vandetanib has been suggested to rely not solely on inhibition of angiogenesis but also on direct attenuation of tumor growth by blockade of its target receptors namely vascular, endothelial growth factor receptor 2 and 3, epidermal growth factor receptor (EGFR) and c-RET expressed on the cancer cells themselves. [9–11]. VEGFR-2 and EGFR receptors have recently been identified as medulloblastoma oncogenes with EGFR gene amplification and overexpression being a marker of poor prognosis [12–16]. We hypothesize that the most aggressively growing medulloblastoma variants characterized by dependency on tumor-angiogenesis and expression of receptor tyrosine kinases represent an attractive target for Vandetanib therapy. However, despite the successful incorporation of Vandetanib into treatment regimes for adult malignancies, assessment of efficacy in pediatric tumor entities is sparse with no preclinical or clinical studies analyzing its use for medulloblastoma treatment yet [17, 18].

Here we present first evidence that Vandetanib has profound cytotoxic activity in pediatric medulloblastoma cell lines derived from high-risk SHH-TP53-mutated and MYC-amplified Non-WNT/Non-SHH medulloblastoma [19–25]. In keeping with the observed anti-neoplastic capacity we demonstrate that Vandetanib treatment attenuates AKT and STAT3 activity, downstream elements of aberrant signaling pathways in medulloblastoma [26–30].

In our latest study we already documented promising *in vitro* and *in vivo* potential for the clinically available PI3K inhibitor GDC-0941 for medulloblastoma therapy [31]. In this follow-up study we show that specifically inhibiting oncogenic receptor tyrosine kinases in combination with downstream elements such as PI3K on which multiple carcinogenic pathways converge might be a rational treatment strategy for aggressive medulloblastoma variants. In this context we observed that the concomitant application of Vandetanib and GDC-0941 resulted in augmented cytotoxicity for MYC-amplified and SHH-TP53-mutated medulloblastoma. In MYC-amplified medulloblastoma we also detected a corresponding reduction of AKT and STAT3 phosphorylation and protein levels compared to single drug treatment. Furthermore, we document that concomitant application of Vandetanib and the standard chemotherapeutic Etoposide leads to enhanced cytotoxicity in comparison to single drug application. Additional inhibition of the PI3K by GDC-0941 further augmented this marked anti-neoplastic efficacy of the Vandetanib-Etoposide combination with de facto no surviving cells in MYC-amplified and SHH-TP53-mutated medulloblastoma. Thus, therapeutic approaches based on inhibition of oncogenic kinases alone or in combination with standard chemotherapeutics might constitute a promising treatment option for medulloblastoma variants with poor clinical outcome to date.

## RESULTS

### In medulloblastoma Vandetanib reduces cell viability in a dose-dependent manner

**Figure 1:**
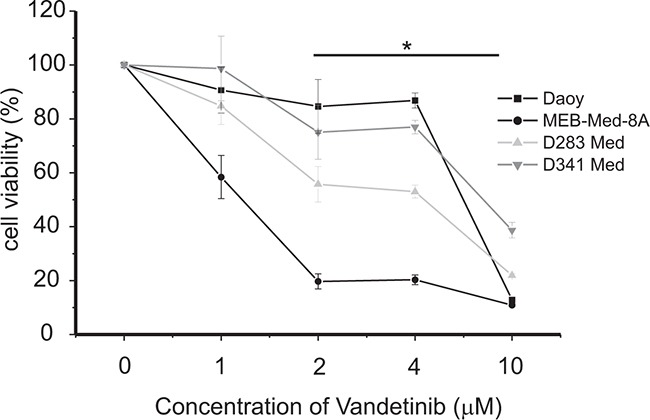
Vandetanib treatment leads to a dose-dependent reduction of medulloblastoma cell viability The cell lines MEB-Med-8A, D283 Med, Daoy and D341 Med were treated with increasing concentrations of vandetanib. The vehicle DMSO served as control. After 48h of drug exposure the cell viability was assessed by means of the MTS assay. Statistically significant differences from DMSO are marked by an asterisk (*p<0.05), the data shown represent four independent experiments.

In a dose-response analysis we evaluated the anti-carcinogenic potency of Vandetanib in Daoy, a cell line modeling SHH-TP53-mutated medulloblastoma, and in the MYC-amplified Non-WNT/Non-SHH medulloblastoma derived cell lines MEB-Med8-A, D283 Med and D341 Med, (Figure 1). At standard growth conditions the cells were treated with Vandetanib concentrations ranging from 1 to 10 μM for 48h. Subsequently the cell viability was determined by MTS assays.

At 2 μM, a concentration corresponding to patient plasma levels [5, 32], Vandetanib reduced cell viability in Daoy by 15±9%, in MEB-Med-8A by 81±3%, in D283 Med by 44±6% and in D341 Med by 25±9% in comparison to control. With regards to Vandetanib susceptibility, MEB-Med-8A and D283 Med were of high responsiveness while Daoy and D341 displayed lower sensitivity. A further decline in cell viability could only be achieved when raising the drug concentration to 10 μM. At this concentration we observed a profound loss in cell viability across all analysed cell lines with a minimum residual cell survival of 10-40%.

### Vandetanib reduces viable cell number and exerts anti-proliferative and pro-apoptotic effects in medulloblastoma cell lines

After 48h of Vandetanib treatment we determined the absolute number of viable cells by flow cytometry and assessed the relative contribution of cell death and proliferation inhibition via combined CFSE-Hoechst33258 stain.

**Figure 2:**
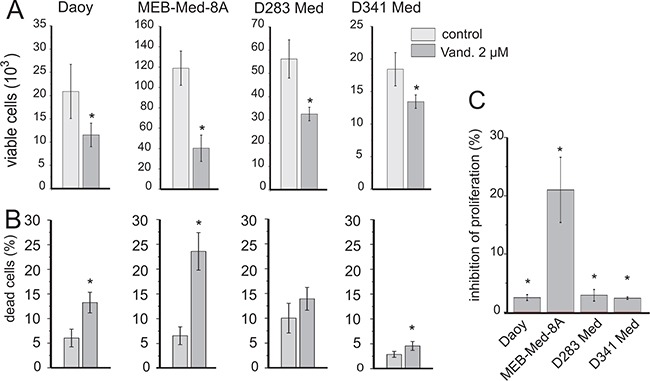
Vandetanib impairs cell viability in medulloblastoma The established paediatric MB cell lines MEB-Med-8A, D283 Med, Daoy and D341 Med were treated with 2 μM of Vandetanib for 48h. The vehicle DMSO served as the control. In a combined flow cytometric cell viability/proliferation assay based on a CFSE/Hoechst 33258 stain, viable cell number **A**. cell death **B**. and inhibition of proliferation **C**. was assessed. All statistically significant differences from DMSO are marked by an asterisk (*p<0.05) **(A-C)**. Inhibition of proliferation was normalized to DMSO **(C)**. The data shown represent four independent experiments.

Exposing the cell lines to 2 μM of Vandetanib over 48h significantly decreased the absolute number of viable cells in Daoy by 35±16%, in MEB-Med-8A by 67±6%, in D283 Med by 41±11% and in D341 Med by 27±9% (Figure 2A). The CFSE-Hoechst33258 stain revealed that this profound loss in absolute cell numbers over a 48h period was predominantly due to the cytotoxic activity of Vandetanib and complemented by a less pronounced anti-proliferative effect (Figure 2B and 2C). At 48h, in Daoy, MEB-Med-8A and D341, 13±2%, 24±4% and 4±1% of dead cells were determined respectively. In D283 Med cell death differed not significantly from control at the assessed time point. With regards to proliferation in MEB-Med-8A a marked reduction of proliferating cells by 21.5% was observed at the 48h time point In contrast in Daoy, D283 Med and D341 Med proliferation in cells surviving after 48h of Vandtanib exposure was only marginally impaired.

### Vandetanib interferes with the clonogenicity of medulloblastoma in a dose-dependent manner

**Figure 3:**
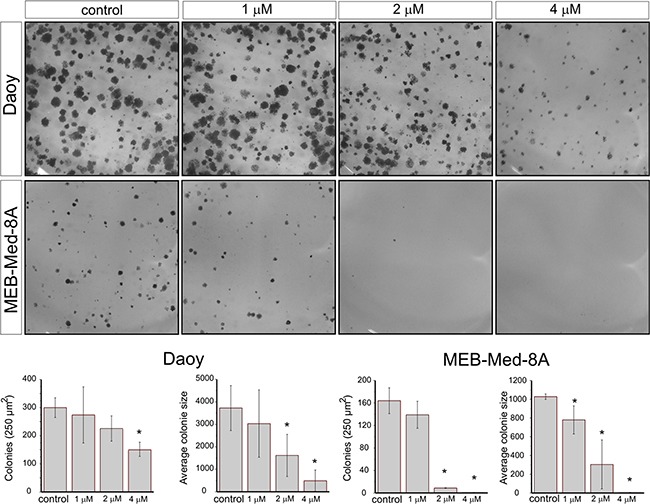
Vandetanib suppresses colony formation of medulloblastoma Daoy and MEB-Med-8A cells were exposed to 1, 2 and 4 μM Vandetanib for 48h. Subsequently the cells were maintained in standard growth medium for 7 days and colony formation and colony size were assessed. Statistically significant differences from control are marked by an asterisk (*p<0.05). The data shown represent five independent experiments.

In view of their differential group affiliation, we chose Daoy and MEB-Med-8A to assess the efficacy of Vandetanib to interfere with medulloblastoma clonogenicity (Figure 3). Treatment of Daoy and MEB-Med-8A with 1, 2 and 4 μM of Vandetanib resulted in a dose-dependent reduction of colony numbers (CN) and average colony size (ACS).

At 2 μM, a concentration typically achieved in patient plasma, MEB-Med-8A showed significant reduction of colony numbers and colony size (CN: 9±0.5 and ACS: 305±250p^2^) in comparison to control (CN: 164±23 and ACS: 1028±29p^2^). At the same concentration in Daoy we only documented a reduction in colony size (1618±900p^2^) compared to control (3723±1000p^2^) but not in colony numbers. Increasing the dose to 4 μM enhanced the observed effects in both cell lines and additionally resulted for Daoy in a profound decline in the number of colonies (173±45) in comparison to control (300±35).

### Vandetanib attenuates medulloblastoma cell migration

**Figure 4:**
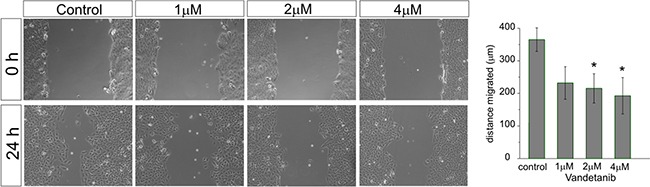
Vandetanib inhibits MB cell migration already at low concentrations After a single scratch was placed in a confluent monolayer of Daoy cells, cell were exposed to increasing concentrations of Vandetanib for 24h. After 24h each scratch was photographed and its width measured. Migration was determined by comparison of the scratch wound size between 0h and 24h. Statistically significant differences from control are marked by an asterisk (*p<0.05). The data shown represent five independent experiments.

We also determined the potential of Vandetanib to inhibit medulloblastoma cell migration at different concentrations (Figure 4). The cell line Daoy was chosen because of its known migratory properties. [33]. After 24 hours at 2 μM, Vandetanib inhibited cell migration significantly (215±45 μm) in comparison to control (365±36 μm). Escalating the dose to 4 μM did not lead to a further impairment of the migratory capacity of Daoy cells.

### Vandetanib and the PI3K inhibitor GDC-0941 display additive anti-neoplastic efficacy in medulloblastoma

**Figure 5:**
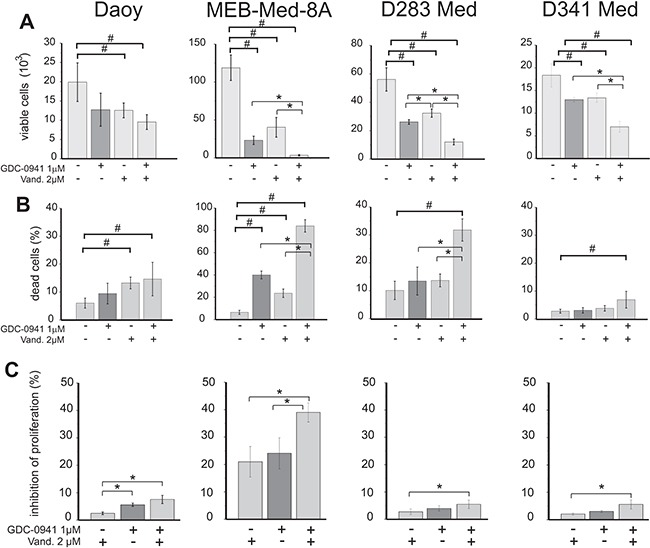
Determination of anti-proliferative and cytotoxic effects of Vandetanib in combination with the PI3K inhibitor GDC-0941 Medulloblastoma cells were treated with 2 μM of Vandetanib or 1 μM of the PI3K inhibitor GDC-0941 alone or in combination for 48h. Viable cell number **A**. cell death **B**. and inhibition of proliferation **C**. was analysed by flow cytometry following a CFSE/Hoechst 33258 stain. DMSO was used as control. Inhibition of proliferation was normalized to control DMSO and all stated values differ significantly (p<0.05) from the control DMSO. Other values tagged with a hashsign also differ significantly from the control (#p<0.05), while all values tagged with an asterisk differ significantly from single drug application (*p<0.05). The data shown represent four independent experiments.

The PI3K/AKT pathway transduces signals of oncogenic receptor tyrosine kinases in medulloblastoma [29, 30]. In this context we have previously shown in *in vitro* and *in vivo* medulloblastoma models that single application of GDC-0941 exerts profound anti-neoplastic activity [31]. We therefore asked whether additional blockade of the PI3K would enhance the cytotoxity of Vandetanib. Here we exposed medulloblastoma cell lines to 2 uM of Vandetinib in combination with 1 uM of the P3IK-antagonist GDC-0941 for 48h. Both drug concentrations correspond to plasma levels found in treated patients [32, 34]. Subsequently the number of viable cell was determined by flow cytometry. In order to differentially assess cell death in relation to proliferation, we applied a CSFE-Hoechst33258 stain (Figure 5).

In the MYC-amplified cell lines the combination of Vandetanib and GDC-0941 resulted in a significantly enhanced reduction of viable cells by 97±1% in MEB-Med-8A, by 78±5% in D283 Med and by 62±1% in D341 Med in comparison to Vandetanib alone with 67±6%, 41±11% and 27±9% and GDC-0941 alone with 81±2%, 53±5% and 29±7% of residual viable cells in MEB-Med-8A, D283 Med and D341 Med respectively. In the SHH-TP53-mutated cell line Daoy we detected on average less residual cells for the drug combination in comparison to single drug application. However, this trend was not significant.

For the MYC-amplified cell lines MEB-Med-8A, D283 Med and D341 Med but not the SHH-TP53-mutated cell line Daoy concomitant exposure to Vandetanib and GDC-0941 led to significantly enhanced cytotoxicity compared to single drug treatment. At 48h after drug treatment we document for the drug combination 84±5% of dead cells in MEB-Med-8A, 32±4% in D283 Med and 7±3% in D341 Med. In contrast Vandetanib single application resulted only in 24±4% of cell death in MEB-Med-8A, 14±2% in D283 Med and 4±1% in D341 Med. In Daoy, D283 Med and D341 Med the effect of Vandetanib single application with 13±2%, 14±2% and 4±1% of dead cells was comparable to the application of GDC-0941 alone with 9±4%, 14±5% and 3±1% of cell death respectively. In MEB-Med-8A with 40±3% cell death 1 μM GDC-0941 displayed more efficacy compared to 2 μM of Vandetanib with 24±4% cell death.

Only in MEB-Med-8A we determined a markedly enhanced inhibitory effect on proliferation with 39±4% when Vandetanib was combined with GDC-0941 in comparison to Vandetanib single treatment with 21±5% inhibition of proliferation. In Daoy, D283 Med and D341 Med proliferation was only marginally decreased by single application of Vandetanib or GDC-0941 at the assessed time point.

### Concomitant application of Vandetanib and GDC-0941 leads to profound reduction of AKT and STAT3 signaling in medulloblastoma cell lines

**Figure 6:**
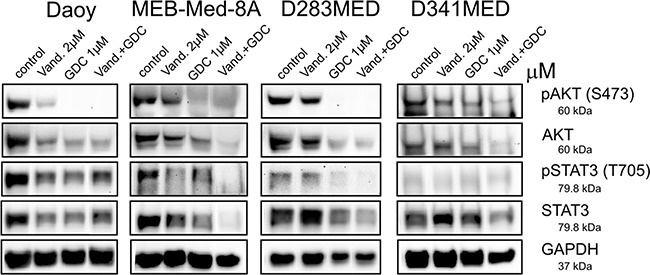
Combinational treatment of Vandetanib and GDC-0941 leads to profound inhibition of STAT3 and PI3K/AKT activation In Daoy, MEB-Med-8A, D283 Med and D341 Med cells were treated with 2 μM of Vandetanib for 48h alone or in combination with 1 μM of GDC-0941 respectively. Total protein levels and phosphorylation status of AKT and STAT3 were determined by Western blot. GAPDH served as loading control.

Aberrant activation of the PI3K/AKT pathway and transcription factor STAT3 is considered critical for medulloblastoma carcinogenesis [26–31]. In keeping with these findings the four investigated medulloblastoma cell lines showed phosphorylation of serine 472 of AKT and tyrosine 705 of STAT3 (Figure 6).

Here we document that in Daoy Vandetanib treatment moderately decreased AKT phosphorylation and protein expression, while in MEB-Med-8A, D283 Med and D341 Med AKT signaling was unaffected by Vandetanib. With regards to STAT3 following treatment with Vandetanib a concomitant decrease in STAT3 phosphorylation and expression was apparent for Daoy and to a greater extent for MEB-Med8-A, but not D283 and D341.

Single treatment of GDC-0941 in Daoy, MEB-Med-8A and D283 Med resulted in complete abrogation of AKT phosphorylation and a marked reduction in AKT protein levels, while in D341 Med residual AKT activity could be detected. STAT3 phosphorylation and protein expression was moderately decreased by GDC-0941 in Daoy, MEB-Med-8A and D283 Med but not in D341 Med.

Application of the Vandetanib-GDC combination enhanced the reduction of AKT phosphorylation and expression only in D341 Med. In Daoy, MEB-Med-8A and D283 Med the already profound suppression of GDC-0941 single application on AKT signaling was not further augmented by the drug combination. With respect to STAT3 activity however, the drug combination resulted in a more pronounced reduction of STAT3 phosphorylation and expression compared to single drug application in MEB-Med-8A, D283 Med and D341 Med. In Daoy in contrast, we could not observe any additional reduction in STAT3 phosphorylation or expression for the combination of Vandetanib and GDC-0941.

### Concomittant application of Vandetanib, GDC-0941 and Etoposide displays additive cytotoxic activity in medulloblastoma

**Figure 7:**
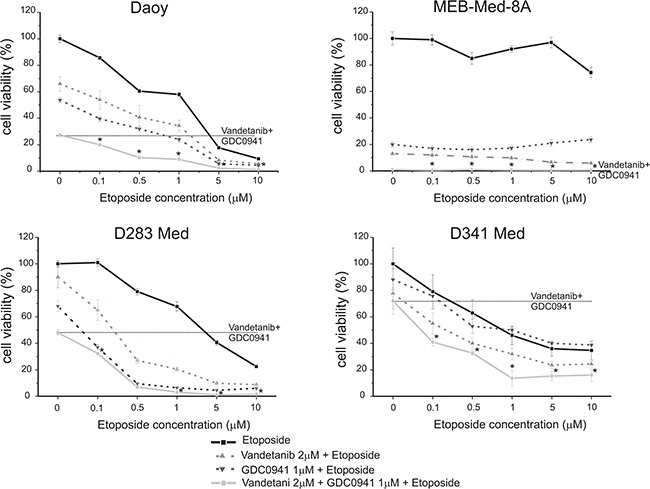
Concomittant application of Vandetanib, GDC-0941 and Etoposide displays additive cytotoxic activity Daoy, MEB-Med-8A, D283 Med and D341 Med were treated with increasing concentrations of the standard chemotherapeutic Etoposide (0.1, 0.5, 1, 5, 10 μM) in combination with 2 μM of Vandetanib and 1 μM of GDC-0941. 1-2 μM of Etoposide corresponds to cerebral spinal fluid levels, while 10 μM of Etoposide corresponds to plasma levels. The vehicle DMSO served as control. After 48 h drug exposure cell viability was assessed by means of the MTS assay. Each experiment was performed in triplicates and repeated four times.

The topoisomerase inhibitor Etoposide is part of the standard treatment regime for paediatric medulloblastoma. We analysed whether concomitant application of Vandetanib and Etoposide at concentrations corresponding to cerebral spinal fluid (1-2 μM) or patient plasma (10 μM) levels results in enhanced anti-neoplastic efficacy [35, 36]. For this purpose we treated medulloblastoma cell lines with Etoposide concentrations ranging from 0.1 – 10 μM in combination with 2 μM of Vandetanib for 48h and determined the cell viability by MTS assay (Figure 7).

In the SHH-TP53-mutated cell line Daoy and the MYC-amplified cell lines D283 Med and D341 Med single application of Etoposide at cerebral spinal fluid levels of 1 μM displayed anti-neoplastic efficacy with 30-60% residual cell viability. In contrast MEB-Med-8A, which also models MYC-amplified tumors, was resistant at 1 μM of Etoposide and displayed only moderate susceptibility to 10 μM Etoposide corresponding to patient plasma levels with 74±4% residual cell viability.

We have previously show additive anti-neoplastic efficacy for the concomitant application of Etoposide and the PI3K-antagonist GDC-0941 in medulloblastoma cell lines [31]. Here we corroborate these results and document superior additive anti-neoplastic efficacy for the Vandetanib-Etoposide combination in comparison to single drug application. In the SHH-TP53-mutated cell line Daoy the anti-neoplastic efficacy of the Vandetanib-Etoposide and GDC-Etoposide combination were similar. In the MYC-amplified cell line D283 Med concomitant application of GDC-0941 and Etoposide displayed superior efficacy compared to concomitant application of Vandetanib and Etoposide. On the opposite in the MYC-amplified cell lines D341 Med and MEB-Med-8A we observed superior efficacy for the Vandetanib-Etoposide combination in comparison to the GDC-Etoposide combination. Of note, in MEB-Med-8A the anti-neoplastic efficacy was predominantly due to kinase inhibitor treatment.

With regards to concomitant application of Vandetanib and GDC-0941 in the SHH-TP53-mutated cell line Daoy we determined in our previous experiments a trend of higher anti-neoplastic efficacy in comparison to single drug application. Here in a cumulative assay determining anti-proliferative and cytotoxic drug effects by means of the metabolic state of the residual cell population this trend could be confirmed. Furthermore, we posed the question, whether inhibition of receptor tyrosine kinases and their downstream signaling molecules could enhance medulloblastoma standard treatment regimes. We treated the investigated cell lines therefore concomitantly with Vandetanib and GDC-0941 in the presence of the chemotherapeutic Etoposide. Indeed, in the SHH-TP53-mutated cell line Daoy as well as in the MYC-amplified cell lines D283 Med and D341 Med the application of the Vandetanib-GDC-Etoposide combination augmented the cytotoxic effect regardless of the applied concentration of Etoposide in comparison to single or dual drug application. In detail, in Daoy and D283 Med at 1 and 5 μM of Etoposide respectively the concomitant application of Vandetanib, GDC-0941 and Etoposide resulted in complete loss of viable cells while in D341 Med at Etoposide concentrations of 1 and 5 μM residual cell viability plateaued 13.6±6 and 15.3±4% respectively. Applying higher Etoposide concentrations in combination with the applied kinase inhibitors did not lead to further enhancement of anti-neoplastic efficacy. In line with above described Etoposide resistancy of MEB-Med-8A concomitant application of Vandetanib and GDC-0941 was sufficient to reduce cell viability completely.

## DISCUSSION

In this study we report that Vandetanib exerts anti-neoplastic activity against a panel of four pediatric medulloblastoma cell lines. MEB-Med-8A, D283 Med and D341 Med exhibit distinct characteristics such as MYC-amplification and occurrence of isochromosome 17 of the most aggressive group of tumors within the Non-WNT/Non-SHH group of medulloblastoma [19–23]. In contrast Daoy displays markers of a subentitiy of SHH-group tumors that is characterized by mutation of tumor suppressor gene p53 (SHH-TP53-mutated) [24, 25]. A most recent report documents that patients suffering from SHH-TP53-mutated medulloblastoma also display extremely poor survival rates [7, 8].

Our analysis details that Vandetanib treatment at concentrations corresponding to patient plasma levels leads to marked loss of viable cells in MYC-amplified and SHH-TP53-mutated medulloblastoma cell lines. In line with the observed anti-tumorigenic efficacy of Vandetanib, transcriptional profiling revealed high mRNA levels of the Vandetanib target structures VEGFR 2 and 3 across all investigated cell lines. In contrast expression of EGFR was only present in Daoy (Supplementary Table 1). Dissecting the observed cumulative anti-neoplastic effect of Vandetanib reveals that the profound cytotoxic activity is complemented by a lower capacity to inhibit proliferation by arresting surviving medulloblastoma cells in G_0_/G_1_-Phase of the cell cycle (Supplementary Figure 1). The cytotoxic capacity of Vandetanib against medulloblastoma is also reflected in attenuated clonogenicity with profound reduction in colony number and size. Beyond cytotoxic and anti-proliferative effects we demonstrate that *in vitro* Vandetanib impairs migration of medulloblastoma cells, a prerequisite for invasion and metastasis. At this point it is noteworthy that inspite of the more narrow target-spectrum, the MKI Vandetanib displays similar *in vitro* efficacy against medulloblastoma as we have previously described for the broad-spectrum MKI Sorafenib and Pazopanib that inhibits amongst numerous other kinases also VEGFR 2 and 3 (Supplementary Table 2). In an orthotopic xenograft modell of MYC-amplified medulloblastoma, Sorafenib and Pazopanib suppressed tumor growth with significantly prolonged survival [28]. This finding is in line with clinical phase I/II trials that document tumor responses of the VEGF antibody bevacizumab in combination with standard chemotherapy in patients suffering from recurrent medulloblastoma [37]. The current investigation therefore corroborates pre-clinical and clinical reports delineating a key role for VEGF signaling in medulloblastoma development and progression that can be exploited for therapy. As it may be inferred that the documented cytotoxic *in vitro* effects for Vandetanib also translate into anti-neoplastic efficacy *in vivo* as shown for the broad-spectrum MKI Sorafenib and Pazopanib, this approach warrants further evaluation [28].

In keeping with the anti-tumorigenic capacity of Vandetanib, we observed a moderate reduction in phosphorylation of the VEGFR 2/3 and EGFR downstream signaling molecule STAT3 in Daoy and MEB-Med-8A. These observations confirm previous investigations delineating the importance of STAT3 pathway activation downstream of aberrant receptor tyrosine kinases signaling for tumorigenesis in medulloblastoma [26–28]. In view of the pronounced cytotoxic activity of Vandetanib in D283 Med and D341 Med, lack of concomitant downregulation of STAT3 phosphorylation indicates that in these cell lines other pathways e.g. mitogen-activated protein kinase pathways as also shown for osteosarcoma might be involved in the integration of oncogenic signals transmitted by Vandetanib target structures [29, 38].

The narrow target profile of Vandetanib in comparison to broad-spectrum anti-angiogenetic MKI such as Pazopanib and Sorafenib results in better safety and tolerability (Supplementary Table 2) [39–42]. This is of particular interest since MKI-triggered side effects such as suppression of haematopoiesis are critical when considering administration of these drugs in combination with chemotherapy, immunotherapy and other targeted agents. At present only few combinations of broad-spectrum MKI with other targeted agents have been reported feasible in terms of safety [43–46]. Whether the more specific inhibitor Vandetanib shows less toxicity when combined with other targeted agents is momentarily under clinical investigation in a phase I trial testing the tolerability and efficacy of Vandetanib in combination with the PI3K/AKT pathway antagonist Everolismus (NCT01582191) in pediatric patients with advanced solid malignancies. Preliminary reports of this phase I study describe good tolerability of the drug combination and evidence of response in heavily pre-treated pediatric patients suffering from sarcoma [47]. Aberrant PI3K/AKT signaling downstream of receptor tyrosine kinases has also been shown critical for the development and progression of medulloblastoma [29, 30]. In this context we documented previously that the clinically available highly specific PI3K inhibitor GDC-0941 displays profound *in vitro* activity and prolonged the life of tumor-bearing animals in an orthotopic xenograft mouse model of most aggressive MYC-amplified human medulloblastoma. [31]. Therefore, we assessed whether additional inhibition of the PI3K by GDC-0941 would further enhance the observed anti-neoplastic effect of Vandetanib. Indeed, here we document that Vandetanib in combination with GDC-0941 displays enhanced cytotoxicity against MYC-amplified and SHH-TP53-mutated tumor modeling cell lines compared to single drug application. In line with the enhanced anti-neoplastic effects of the Vandetanib-GDC-combination our findings demonstrate in MYC-amplified medulloblastoma a further reduction in AKT and STAT3 activity respectively in comparison to single drug application. This loss in activity was not solely due to inactivation of upstream kinases but also to reduction of the respective protein levels. Absence of AKT and STAT3 proteins would also counteract other oncogenic kinase signaling via these pathways. In context the SHH-TP53-mutated cell line Doay we did not detect a difference in AKT and STAT3 protein levels for the Vandetanib-GDC combination in comparison to single drug application. However, since only one SHH-TP53-mutated cell line was investigated and Vandetanib alone diminished besides STAT3 also AKT activity in the respective cell line, this result should be regarded with caution. With respect to the observed reduction in AKT activity might be of note that the SHH-TP53-mutated cell line expresses not only the Vandetanib target structures VEGFR 2 and 3 as the MYC-amplified cell lines but also EGFR. In breast cancer cell lines inhibition of VEGFR and PI3K signaling by the multi-kinase inhibitor Sorafenib leads to a reduction of MYC activity [48]. Whether Vandetanib or GDC-0941 also exert anti-neoplastic efficacy by suppressing MYC activity warrants further evaluation.

Given the recent publications on medulloblastoma subgrouping, the next step with regards to therapy is to adapt the existing treatment regimes for high risk medulloblastoma variants [6, 8, 49]. In this context our study documents besides anti-neoplastic efficacy of the standard chemotherapeutic Etoposide in MYC-amplified as well as SHH-TP53-mutated medulloblastoma cell lines an additive anti-tumorigenic efficacy for the Etoposide-Vandetanib combination in comparison to single drug application (Figure 7). Our previous publication documented also additive efficacy against the same panel of medulloblastoma cell lines for the concomitant application of Etoposide and the PI3K inhibitor GDC-0941 [31]. Here we corroborate these results and show that the Vandetanib-Etoposide as well as the GDC-Etoposide combination display similar efficacy in SHH-TP53-mutated and MYC-amplified cell lines.

Of note compared to single or dual drug application, concomitant application of all three drugs reduces residual cell viability to negligible levels even at very low doses of etoposide. This is of considerable interest since patients undergoing medulloblastoma therapy often suffer from neurological sequelae due to chemotherapeutic mediated long-term side-effects [50]. Therefore incorporation of targeted agents into standard treatment regimes could also positively affect the life-quality of patients significant reduction of applied chemotherapeutic dosage.

Of particular interest MEB-Med-8A, a cell line refractory to both etoposide as well as cisplatin (data not shown), was still highly sensitive to single treatment with Vandetanib and GDC-0941 respectively. Moreover, concomitant application of Vandetanib and GDC-0941 resulted in de facto complete loss of cell viability. Whether this finding, although promising, can be translated to refractory tumors therapy in patients needs further evaluation.

The development of rational and personalized cancer therapy depends on the identification of valid biomarkers. Expression of target structures might serve as markers for the integration of TKI such as Vandetanib into standard treatment regimes of medulloblastoma. However, in other cancers microRNA expression patters are discussed as promising indicators of eligibility for patient treatment with Vandetanib [51].

In general, our findings underscore the notion that combined inhibition of specific oncogenic tyrosine kinases together with important downstream signaling nodes - such as the PI3K that integrates signals of multiple oncogenic kinases - can enhance anti-neoplastic efficacy in combination with standard medulloblastoma chemotherapeutics.

In conclusion, our study demonstrates that Vandetanib, a clinically available angiogenesis inhibitor, displays direct anti-neoplastic activity against pediatric medulloblastoma cell lines modeling the clinically most aggressive SHH-TP53-mutated and MYC-amplified tumor variants. Furthermore, our study documents that combined application of Vandetanib and GDC-0941, a clinically available PI3K/AKT pathway inhibitor, results in fortified anti-neoplastic responses against SHH-TP53-mutated and MYC-amplified medulloblastoma. Furthermore, we delineate that Vandetanib in combination with the standard chemotherapeutic Etoposid displays additive anti-neoplastic efficacy that can be further enhanced by PI3K inhibition. Therefore, our findings provide a rational to further investigate Vandetanib alone, in combination with PI3K/AKT pathway inhibitors and standard chemotherapeutics for the treatment of medulloblastoma variants that despite of intensive multi-modality treatment are currently still associated with poor prognosis.

## MATERIALS AND METHODS

### Reagents and antibodies

Vandetanib and GDC-0941 were obtained from LC Laboratories. The primary antibody pSTAT3 (TYR705, D3A7), STAT3 (124H6), pAKT (Ser473), AKT (11E7) and GAPDH (D18H11) were purchased from Cell Signaling while secondary antibodies were purchased from Dianova. Carboxyfluoreszein-Succinimidyl Ester (CFSE) was purchased from Invitrogen, while Hoechst 33258 was provided by Sigma.

### Cell culture

The human medulloblastoma cell lines, Daoy (HTB 186), D283 Med (HTB-185) and D341 Med (HTB-187) were obtained from American Type Culture Collection (ATCC). The medulloblastoma cell line, MEB-Med-8A, was generated by Prof. T. Pietsch. The medulloblastoma cell lines Daoy, D283 Med and MEB-Med-8A were maintained in complete medium, namely Dulbecco's Modified Eagle Medium (DMEM, PAA) with L-glutamine supplemented with 1 mM sodium pyruvate (PAA), 1% penicilline/streptomycine (Invitrogen) and 10% fetal bovine serum (FBS, Invitrogen). The medulloblastoma cell line D341 Med was maintained in DMEM with L-glutamine supplemented with 1 mM sodium pyruvate, 1 % penicilline/streptomycine and 10 % Human Serum (HS, PAA).

### Cell viability assay

Cell viability was assessed with CellTiter 96 Aqueous One Solution Cell proliferation Assay (Promega) that contains 3-(4,5-dimethylthiazol-2-yl)-5-(3- carboxymethoxyphenyl)-2-(4-sulfophenyl)-2H-tetrazolium (MTS). To ensure a linear growth curve over 48h for assessment of MKI-effects, each well of 96-well plates was seeded with 2.5×10^3^ Daoy, 6×10^3^ MEB-Med-8A, 10^4^ D283 Med and 10^4^ D341 Med cells respectively. After overnight culture in complete medium, the cells were treated with 1, 2, 4 and 10 μM of Vandetanib alone or in combination with 0.1, 0.5, 1, 5 and 10 μM of etoposide and 1 μM of GDC-0941. The vehicle Dimethylsulfoxid (DMSO) served as control. After 48h of MKI treatment, MTS was added according to the supplier's protocol and the absorbance was measured at 490 nm using an ELISA plate reader (Victor^2^ Wallac, Perkin Elmer). Cell viability was calculated in percent of control.

### Combined proliferation and cell death assay

Medulloblastoma cells were stained with CFSE according to the instructions of the supplier. Daoy, MEB-Med-8A, D283 Med and D341 Med cells were seeded in 6-well cell culture dishes in complete medium. After overnight culture, the cells were treated with 2 μM of Vandetanib for 48h. Thereafter floating and attached cells were collected, resuspended in 200 μl, stained with Hoechst33258 and analysed by flow cytometry. The number of viable cells was assessed for 120 sec in same volume and at constant speed. Proliferation was traced by CFSE staining and normalized to the control DMSO, while cell death was determined by Hoechst33258 staining.

### Cell migration assay

The *in vitro* scratch assay was performed as described by Liang et al. [52]. Briefly, Daoy cells were plated in 12-well cell culture dishes. The cells were allowed to adhere and spread for 12h at 37 °C. The confluent monolayer was scratched in a straight line with a p200 pipette tip. The debris was removed and the cells were then incubated with Vandetanib. The vehicle DMSO served as control. After 24h of treatment, migration of cells into the “wound” was photographed at 10x magnification (Nikon Eclipse TiS inverted microscope attached to a CCD monochrome camera DS 2M). The distance of migration was analyzed by means of NIS-Elements Imaging Software.

### Immunoblotting analysis

A total protein concentration of 25 μg derived from medulloblastoma cell lines was separated by SDS-polyacrylamide gel electrophoresis and transferred to nitrocellulose membranes (Bio-Rad). The membranes were blocked for 1h at RT in 1x Tris-buffered saline containing 0.1% tween-20 (TBST) supplemented with 5% BSA. Thereafter, the membranes were incubated with the primary antibodies overnight at 4°C and subsequently with the respective secondary antibody for 1h at room temperature. Immunoreactivity was detected by chemiluminescence and quantified by means of a ChemiDoc XRS Imaging System (Bio-Rad).

### Colony formation assay

The cell lines Daoy (200 cells/well) and MEB-Med-8A (1000 cells/well) were plated in six well cell culture dishes. The cells were allowed to adhere and spread properly for 12h at 37 °C. Thereafter the cells were exposed to 1, 2 and 4 μM of Vandetanib. After 48h of exposure the cells were washed with standard medium to remove any trace of the inhibitor and cultured for another week. Colony numbers, and colony size was assessed by IMAGEJ. Particles smaller than 40 pixel^2^ were excluded from the analysis since these represented stain artefacts, cell detritus or non-proliferating single cells.

### Statistical analysis

The two-sided Student's *t*-test was applied to determine statistical significance between groups. p<0.05 (*), was considered as statistically significant. Values stated within text and figures represent mean ± standard deviation.

## SUPPLEMENTARY MATERIALS FIGURES AND TABLES



## Abbreviations

ATCC: American Type Culture Collection
CFSE: carboxyfluoreszein-succinimidyl ester
DMEM: Dulbecco's modified eagle medium
DMSO: dimethylsulfoxid
EGFR: epidermal growth factor receptor
M: molar
MKI: multi-kinase Inhibitor
MTS: 3-(4,5-dimethylthiazol-2-yl)-5-(3-carboxy-methoxyphenyl)-2-(4-sulfophenyl)-2H-tetrayolium
PI3K: phosphoinositide-3-kinase
STAT3: signal transducer and activator of transcription 3
VEGFR: vascular endothelial growth factor receptor.
