# Supplementary material for: The anti-neoplastic activity of Vandetanib against high-risk medulloblastoma variants is profoundly enhanced by additional PI3K inhibition
Source: Oncotarget. 2017 Jan 31;8(29):46915–27. doi: 10.18632/oncotarget.14911 (PMC5564532; doi:10.18632/oncotarget.14911)
Supplement: Supplementary file 1 [file oncotarget-08-46915-s001.pdf]

The anti-neoplastic activity of Vandetanib against high-risk medulloblastoma variants is profoundly enhanced by additional PI3K inhibition

SUPPLEMENTARY MATERIALS AND METHODS

Cell cycle analysis

Daoy (2×10^5/well) and MEB-Med-8A (3×10^5/well) cells respectively were plated in 6-well cell culture dishes. After 48h of treatment with 1, 2 and 4 µM of Vandetanib the cells were exposed to 16 nM Hoechst 33342 and incubated for 45 min at 37°C. Both floating and attached cells were harvested and analyzed by flow cytometry (Navious, Beckman Coulter). Dead cells were stained by Propidium Iodid (PI). After gating on live cells, single cells were gated using width and area parameters from Hoechst 33342. The area parameter histogram was used to determine the percentage of cells in G_1, S and G_2M phases.

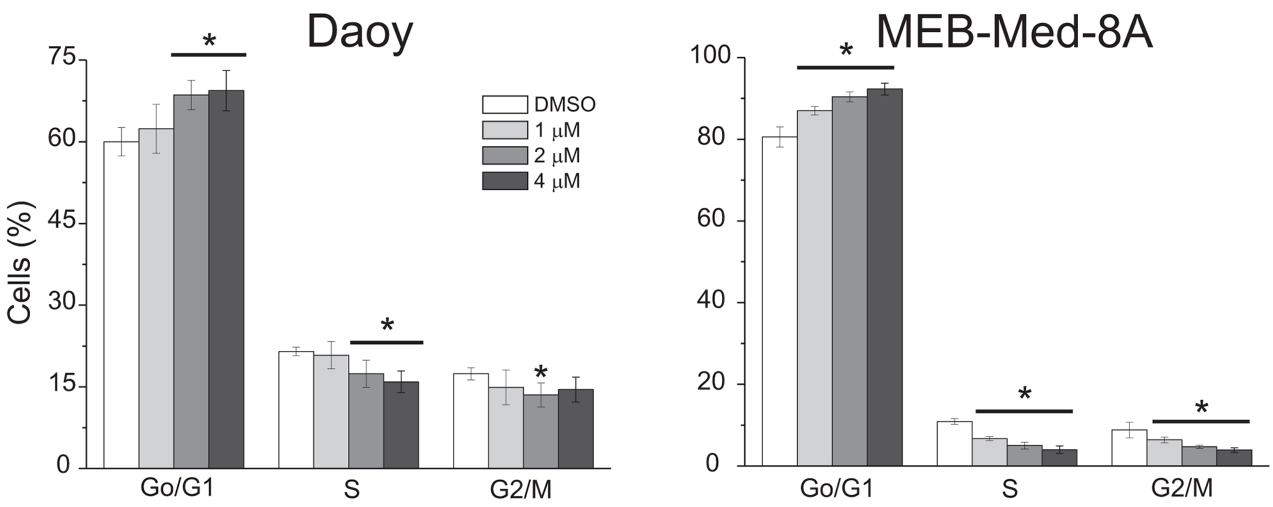

Supplementary Figure 1: Vandetanib induces a G_0/G_1-phase cell cycle arrest in medulloblastoma. Daoy and MEB-Med8A cells were treated with increasing concentrations of Vandetanib (1, 2 and 4 μM) for 48h. Subsequently cell cycle distribution was determined by Hoechst 33342 staining. The vehicle DMSO served as control. Statistically significant differences from control are marked by an asterisk (*p<0.05). The data shown represent five independent experiments.

Supplementary Table 1: Inhibitory concentrations (IC50 in nmol) for MKI targets [1, 2, 3]

Drug	VEGFR-1	VEGFR-2	VEGFR-3	EGFR	c-RET	PDGFR-α	PDGFR-β	c-kit
Vandetanib	>1000	40	110	50	130	>1000	>1000	>10000
Sunitinib	10	10	10	>10000	37	5	10	13
Sorafenib	57	90	20	>10000	2	50	50	68

Protein binding 95-99%

Supplementary Table 2: Expression of Vandetanib target proteins in medulloblastoma cell lines

Receptor	MEB-Med-8a	D283 Med	Daoy	D341 Med
VEGFR-2 *	+	+++	+++	+
VEGFR-3 *	++++	+++	+++	+++
EGFR **	-	-	++++	-

(- not detectable, + = low; ++++ = high)

REFERENCES

1. Marmé D, Norbert F. (2008). Tumor Angiogenesis Basic Mechanisms and Cancer Therapy.
2. Gross-Goupil M1, François L, Quivy A, Ravaud A. Axitinib: a review of its safety and efficacy in the treatment of adults with advanced renal cell carcinoma. Clin Med Insights Oncol. 2013; 7:269-277.
3. Kumar R1, Crouthamel MC, Rominger DH, Gontarek RR, Tummino PJ, Levin RA, King AG. Myelosuppression and kinase selectivity of multikinase angiogenesis inhibitors. Br J Cancer. 2009; 101:1717-1723.
